# Short-term Changes in Aortic Regurgitation after Percutaneous Mitral Valvuloplasty

**Published:** 2013-03-15

**Authors:** Sedigheh Saedi, Mona Heidarali, Tehereh Saedi, Homman Bakhshandeh Abkenar, Mohammad Ali Sadr-Ameli

**Affiliations:** 1 Department of Cardiology, Rajaei Cardiovascular, Medical and Research Centre, Tehran University of Medical Sciences, Tehran, IR Iran

**Keywords:** Percutaneous, Mitral, Valvuloplasty, Mitral Stenosis, Aortic Regurgitation

## Abstract

**Objectives:**

The aim of this study was to evaluate the short- term effects of percutaneous mitral valvuloplasty (PMV) on coexisting AR.

**Methods:**

Clinical, echocardiographic and catheterization data from hospital records of a total of 327 patients with rheumatic mitral stenosis who underwent PMV at a tertiary centre were retrospectively reviewed and aortic regurgitation changes 48 hours post PMV was recorded.

**Results:**

The study population consisted of 282 females and 45 males. Mean age at the time of intervention was 47.13±11 years. Before PMV, 142 (43.3%) patients had no AR, 124 (37.9%) had mild AR and 61 (18.7%) had moderate AR. There was no change in AR severity in post- PMV follow-up. AR progression after PMV and during the follow-up was not significant and there was no increase in the need for aortic valve replacement (AVR) procedures.

**Conclusions:**

Our findings indicated that a considerable number of patients with rheumatic mitral stenosis had concurrent AR. At the time of PMV concomitant AR does affect procedural success and is not associated with inferior outcomes. Patients with moderate degrees of AR remain good candidates for PMV.

## 1. Background

Percutaneous mitral valvuloplasty (PMV) was first described by Inoue et al and has since been widely used to treat mitral stenosis (MS) (1, 2). PMV is recommended in patients with moderate to severe MS who are symptomatic or have pulmonary hypertension hypertension with favorable valve morphology, but no left atrial thrombus (3). About half of the patients with rheumatic MS have some degree of aortic regurgitation (AR) (4). However; it is not well established as to whether AR severity changes after PMV procedures or if these patients need aortic valve (AV) interventions earlier or with increasing frequency. Some studies have postulated that relieving MS may increase the amount of AR, which may prove to be an intolerable burden on the LV or become so severe that necessitates aortic valve replacement (AVR) (5). After PMV for severe MS, it might be expected that AR would increase significantly, mostly due to enhanced blood flow through previously diseased AV and a need for AVR at an earlier than expected time. However, this has not been confirmed by some natural history studies (6-8).

Most previous studies have evaluated patients with mild AR and very few have been done in patients with moderate or severe AR (4)and altogether little is known on the natural history of AR in patients after MV interventions (6, 9). To resolve this issue and given the high prevalence of rheumatic MS in our country and insufficient studies performed in this area, despite the high rates of PMVs performed, we sought to evaluate the short- term effects of PMV on AR progression.

## 2. Materials and Methods

This retrospective study comprised 327 patients who underwent PMV at a tertiary centre between 1994 and 2007. Patients with available data regarding the AR degree of pre-PMV were included in the analysis. The clinical and echocardiographic data were acquired from hospital records. All the patients had undergone transthoracic echocardiography (TTE) and transesophageal echocardiography (TEE), and atrial size, pulmonary artery pressure, presence of LA thrombus, and MR were recorded. Patients with moderate to severe MS who had a valve area <1cm²/m ² of the body surface area and suitable valve morphology (MV score < 9) with no or mild mitral regurgitation (MR) and no left atrial clot were selected for PMV. All the procedures were performed via the Inoue method .Balloons were selected according to patient’s height and body surface area.

Hemodynamic parameters, left and right atrial pressures, and gradient changes were recorded during PMV. Mitral valve area, MR, AR, tricuspid regurgitation severity, LVEF, and left atrial diameter were measured via TTE and TEE before the procedure. All the patients underwent TTE 48 hours after PMV.

Color Doppler study and effective regurgitant orifice area, vena contracta width and pressure half time were used to define the quality and quantity of AR. Based on these findings, AR was classified into three groups of none, mild and moderate AR(6, 10). None of selected patients had aortic stenosis. Left atrial diameter was measured from 2D images. The patients’ heart rhythms, based on the recorded ECGs, were grouped into sinus rhythm or AF before and after PMV. Demographic and clinical variables, including age, sex, and distribution of the data according to age and sex were recorded. A pregnant woman who suffered from critically severe MS and severe AR also underwent successful PMV but was excluded from the study population. Data were collected assigning a personal code to each patient.The institutional ethics committee approved the research protocol.

### 2.1 Statistical analysis

The continuous variables were expressed as mean±SD, and the categorical variables as percentages. The *t*-test was used for the interval variables and chi-square test for the categorical variables. Wilcoxon signed rank test was used to assess the relationship between AR before and after PMV.All the statistical tests were two-sided using α<0.05 level of significance. The data were processed and analyzed using SPSS 15 version.

## 3. Results

The mean age of patients was 47.13±11 years (Table 1). The study population consisted of 282 (86.3%) females.

AR was measured before the procedure via echocardiography; and of a total of 327 patients, 142 (43.2%) had no AR, 124 (37.9%) had mild AR and 61(18.7%) had moderate AR. Echocardiographic evaluation 48 hours post-PMV showed unchanged aortic regurgitation severity in all patients. Follow-up data showed that after PMV, None of our patients had progressed to severe AR. No patient underwent AVR .

The mean of the EF was 52.5±4.3. There was a statistically significant relationship between EF and AR (*P*=0.02).There was also a significant relationship between the EF and rhythm (*P*=0.01), and the patients with sinus rhythm showed a higher mean of EF compared to those with atrial fibrillation(52.9±4.1 and 51.3±4.9 respectively). There was no significant relationship between rhythm and AR severity before PMV (*P*=0.2).

After adjusting for age and sex as the confounding factors, PMV had no effect on the EF. The mean of the EF before and after PMV was not significantly different between the male and female patients.

**Table 1.** Baseline Characteristics Grouped According to AR Severity of Pre-PMV

	Total (n=327 )	Non AR (n=142)	mild AR (n=124)	mod AR (n=61)	*P value[Table-fn fn4997]*
Female sex	282(100%)	122(43.3%)	109(38.7%)	51(18.1%)	0.8
Age(yr)	47.1±10.9	46.6±11.1	47.4±11.4	47.7±9.5	0.7
AF	100%	35.4%	41.8%	22.6%	0.2
Sinus rhythm	100%	45.9%	39.7%	14.4%	0.2
EF	52.5±4.3	52.8±3.8	51.7±4.8	53.4±4.1	0.02
LA diameter	4.6±0.6	4.5±0.6	4.7±0.5	4.5±0.7	0.1

Values are expressed in mean±SD or n (%)

## 4. Discussion

Rheumatic heart disease (RHD) continues to be a common health problem in the developing world, causing morbidity and mortality among children and adults (11). Concurrent involvement of both MV and AV has been reported in one third to one half of patients with RHD (7, 10). Patients with severe AR and concomitant MS are usually not referred for PMV. On the other hand, combined aortic and mitral valve replacement is usually associated with higher procedural risks and poorer long-term survival than is the replacement of either of the two valves (12). In our study, more patients had moderate AR before PMV compared to previous studies(4).

According to this study, we think PMV could be beneficial even in patients with severe AR who do not yet serve the true indication of AVR. Future studies might better clarify this point.

Older patients were more likely to have atrial fibrillation (AF) before and after PMV. These findings are similar to those in some other studies (4, 13).Most patients had sinus rhythm before PMV which was probably due to the selection of lower-risk patients in the earlier stages of MS for PMV. These groups of patients were younger and had a smaller left atrial size, higher EF, and effective valve area after PMV. In patients who had AF rhythm, before and after PMV, the left atrial size was larger, correlated with poorer clinical outcomes and valvular morphology (4, 13).

Although there was no significant relationship between the patients’ rhythm and AR before and after PMV, it seems that the patients without AR were more likely to have sinus rhythm, which may be due to milder forms of the disease.

As pointed in the results section, no post procedure AVR was performed as a result of increased AR severity.

## 5. Conclusion

 Our findings indicate that among patients with rheumatic MS and planned PMV, a considerable number have concomitant aortic regurgitation. Based on these results, we therefore suggest that PMV is an effective treatment for patients with MS and concurrent trivial to moderate AR and is not associated with inferior outcomes.

**Limitations:** In our study, most of the patients had mild or moderate AR and it was not possible to perform a comprehensive assessment of patients with severe AR. One patient with severe AR before PMV tolerated the procedure well, but we do not have corresponding long-term follow- up data.

## References

[1] Inoue K, Owaki T, Nakamura T, Kitamura F, Miyamoto N (1984). Clinical application of transvenous mitral commissurotomy by a new balloon catheter.. J Thorac Cardiovasc Surg..

[2] Tops LF, Kapadia SR, Tuzcu EM, Vahanian A, Alfieri O, Webb JG (2008). Percutaneous valve procedures: an update.. Curr Probl Cardiol..

[3] Bonow RO, Carabello BA, Chatterjee K, de Leon AC, Jr., Faxon DP, Freed MD (2008). 2008 Focused update incorporated into the ACC/AHA 2006 guidelines for the management of patients with valvular heart disease: a report of the American College of Cardiology/American Heart Association Task Force on Practice Guidelines (Writing Committee to Revise the 1998 Guidelines for the Management of Patients With Valvular Heart Disease): endorsed by the Society of Cardiovascular Anesthesiologists, Society for Cardiovascular Angiography and Interventions, and Society of Thoracic Surgeons.. Circulation..

[4] Sanchez-Ledesma M, Cruz-Gonzalez I, Sanchez PL, Martin-Moreiras J, Jneid H, Rengifo-Moreno P (2008). Impact of concomitant aortic regurgitation on percutaneous mitral valvuloplasty: Immediate results, short-term, and long-term outcome.. Am Heart J..

[5] Runco V, Levin H, Wooley C, Booth R (1965). Aortic regurgitation: Angiography study of its natural history following mitral valvotomy and its effect on post operative prognosis.. Circulation..

[6] Ha JW, Choi SH, Chang BC, Nam CM, Jang Y, Chung N (2002). Is prophylactic aortic valve replacement indicated during mitral valve surgery for mild to moderate aortic valve disease?. Ann Thorac Surg..

[7] Namboodiri N, Remash K, Tharakan JA, Shajeem O, Nair K, Titus T (2009). Natural history of aortic valve disease following intervention for rheumatic mitral valve disease.. J Heart Valve Dis..

[8] Padial LR, Oliver A, Vivaldi M, Sagie A, Freitas N, Weyman AE (1997). Doppler echocardiographic assessment of progression of aortic regurgitation.. Am J Cardiol..

[9] Choudhary SK, Talwar S, Juneja R, Kumar AS (2001). Fate of mild aortic valve disease after mitral valve intervention.. J Thorac Cardiovasc Surg..

[10] Spagnuolo M, Kloth H, Taranta A, Doyle E, Pasternack B (1971). Natural history of rheumatic aortic regurgitation. Criteria predictive of death, congestive heart failure, and angina in young patients.. Circulation..

[11] Eisenberg MJ (1993). Rheumatic heart disease in the developing world: prevalence, prevention, and control.. Eur Heart J..

[12] Vaturi M, Porter A, Adler Y, Shapira Y, Sahar G, Vidne B (1999). The natural history of aortic valve disease after mitral valve surgery.. J Am Coll Cardiol..

[13] Maatouk F, Betbout F, Ben-Farhat M, Addad F, Gamra H, Ben-Hamda K (2005). Balloon mitral commissurotomy for patients with mitral stenosis in atrial fibrillation: ten-year clinical and echocardiographic actuarial results.. J Heart Valve Dis..

